# Supplementary material for: Deciphering microbial interactions in synthetic human gut microbiome communities
Source: Mol Syst Biol. 2018 Jun 21;14(6):e8157. doi: 10.15252/msb.20178157 (PMC6011841; doi:10.15252/msb.20178157)
Supplement: Supplementary file 1 — Appendix [file MSB-14-e8157-s001.pdf]

Appendix for

Deciphering microbial interactions in synthetic human gut microbiome communities

Ophelia S. Venturelli*[1], Alex C. Carr[+5], Garth Fisher[+5], Ryan H. Hsu[2], Rebecca Lau[5], Benjamin P. Bowen[5], Susan Hromada[1], Trent Northen[5] & Adam P. Arkin[2345]

[1]Department of Biochemistry, University of Wisconsin-Madison, Madison, WI
[2]California Institute for Quantitative Biosciences, University of California Berkeley, Berkeley CA
[3]Department of Bioengineering, University of California Berkeley, Berkeley CA
[4]Energy Biosciences Institute, University of California Berkeley, Berkeley CA
[5]Environmental Genomics and Systems Biology, Lawrence Berkeley National Laboratory, Berkeley CA

Table of Contents

Appendix Figure S1_________2
Appendix Figure S2_________3
Appendix Figure S3_________4
Appendix Figure S4_________5
Appendix Figure S5_________5
Appendix Figure S6_________6
Appendix Figure S7_________7
Appendix Figure S8_________8
Appendix Figure S9_________8
Appendix Figure S10_________9
Appendix Figure S11_________10
Appendix Figure S12_________10
Appendix Figure S13_________11
Appendix Figure S14_________12
Appendix Figure S15_________12
Appendix Figure S16_________13
Appendix Figure S17_________14
Appendix Figure S18_________14
Appendix Figure S19_________15
Appendix Figure S20_________15
Appendix Figure S21_________16
Appendix Figure S22_________17
Appendix Figure S23_________18
Appendix Figure S24_________19
Appendix Figure S25_________20
Appendix Figure S26_________20
Appendix Figure S27_________20
Appendix Figure S28_________20
References_________21

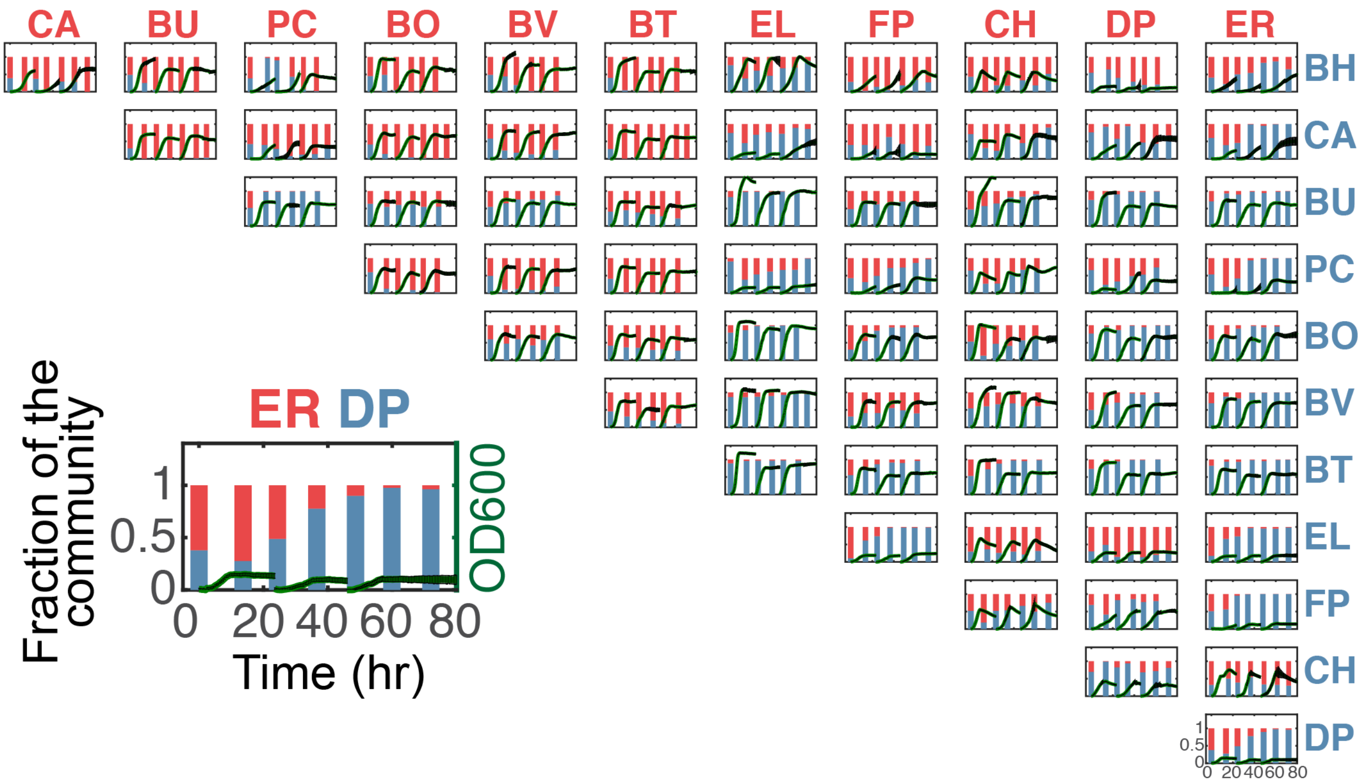

Appendix Figure S1

Relative abundance and total biomass (OD600) measurements as a function of time of all pairwise assemblages inoculated at approximately equal initial species proportions based on OD600 values. Each subplot shows relative abundance (left) and OD600 (right) of the community as a function of time. Error bars represent 1 s.d. from the mean of at least three biological replicates. Species include: *Collinsella aerofaciens* (CA), *Bacteroides uniformis* (BU), *Prevotella copri* (PC), *Bacteroides ovatus* (BO), *Bacteroides vulgatus* (BV), *Bacteroides thetaiotaomicron* (BT), *Eggerthella lenta* (EL), *Faecalibacterium prausnitzii* (FP), *Clostridium hiranonis* (CH), *Desulfovibrio piger* (DP), *Eubacterium rectale* (ER) and *Blautia hydrogenotrophica* (BH).

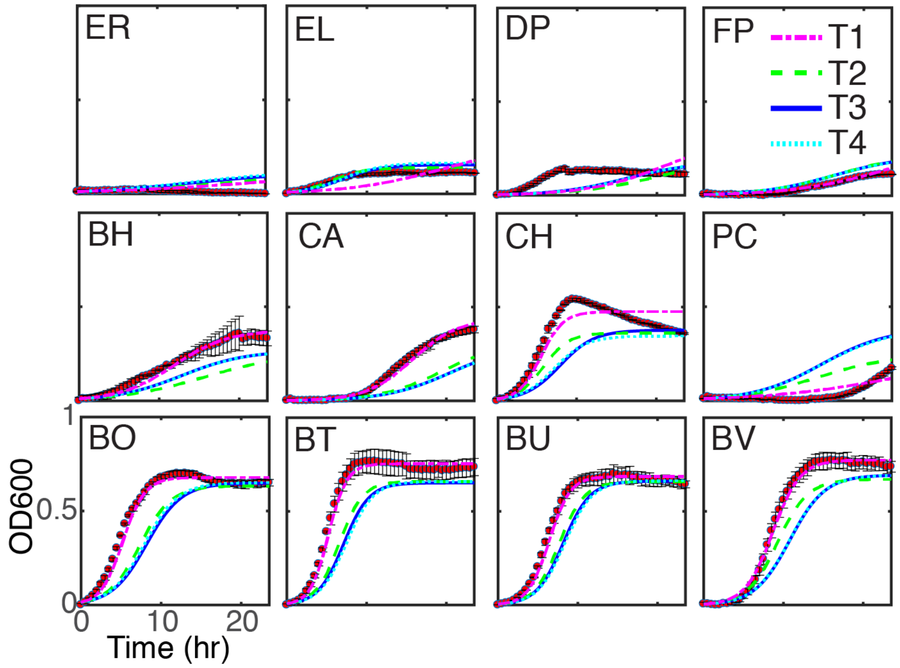

Appendix Figure S2
Growth responses of monospecies as a function of time. Each subplot shows absorbance at 600 nm (OD600) as a function of time for single species. Red circles denote data points. Lines represent gLV model fits trained on T1-T4. Error bars denote 1 s.d. from the mean of three biological replicates.

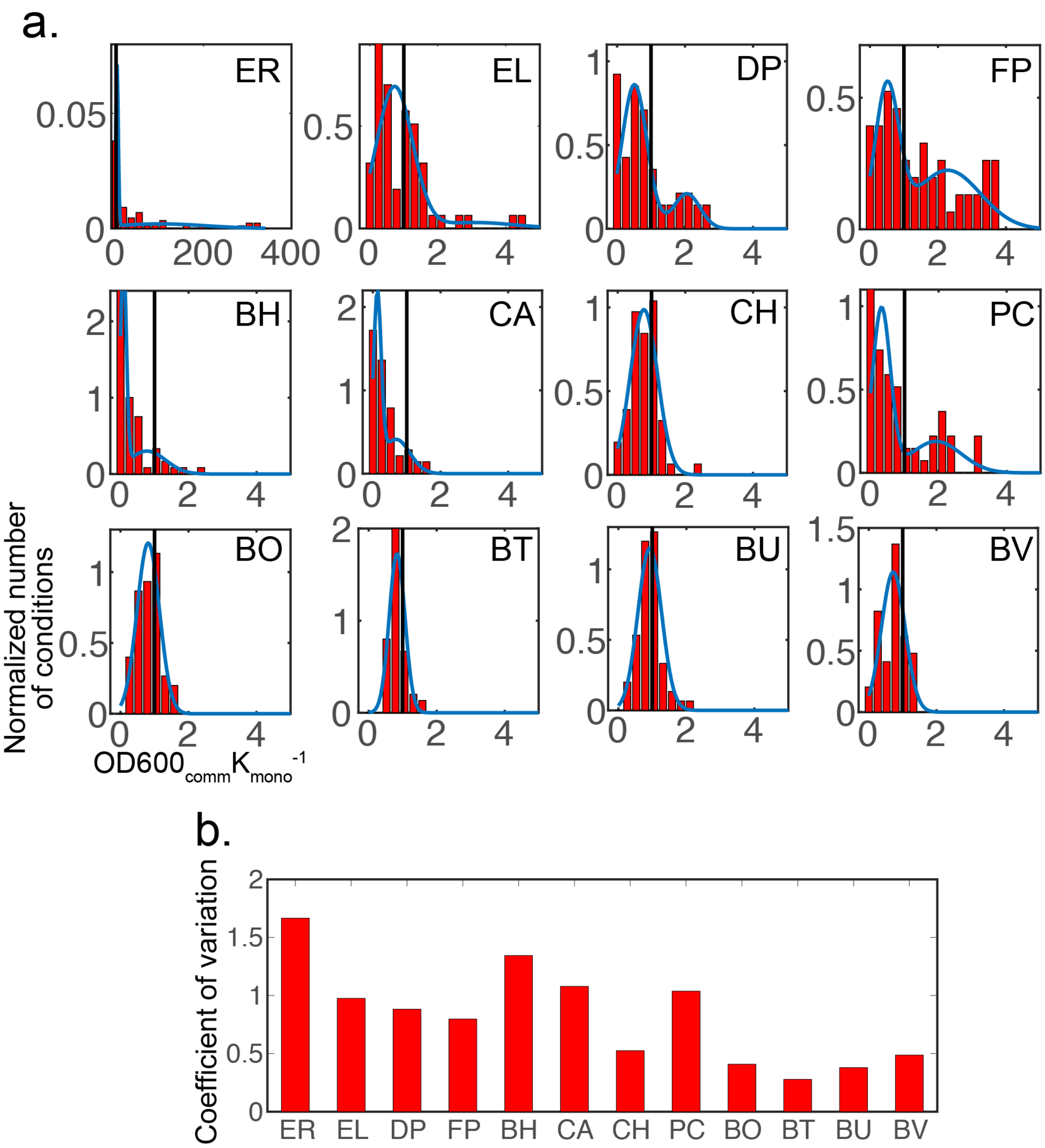

Appendix Figure S3

Absolute species abundance distributions based on OD600 and relative abundance of each species across all pairwise assemblages in PW1. (a) Each subplot shows the absolute abundance ($OD600_{comm}$) divided by the monospecies OD600 value at 23.5 hr (K_{mono}). The y-axis represents the probability density function. Blue and black lines represent Gaussian mixture model fits to the data and x = 1, respectively. (b) Coefficient of variation of absolute abundance distributions divided by the monospecies OD600 value at 23.5 hr ($OD600_{comm} K_{mono}^{-1}$).

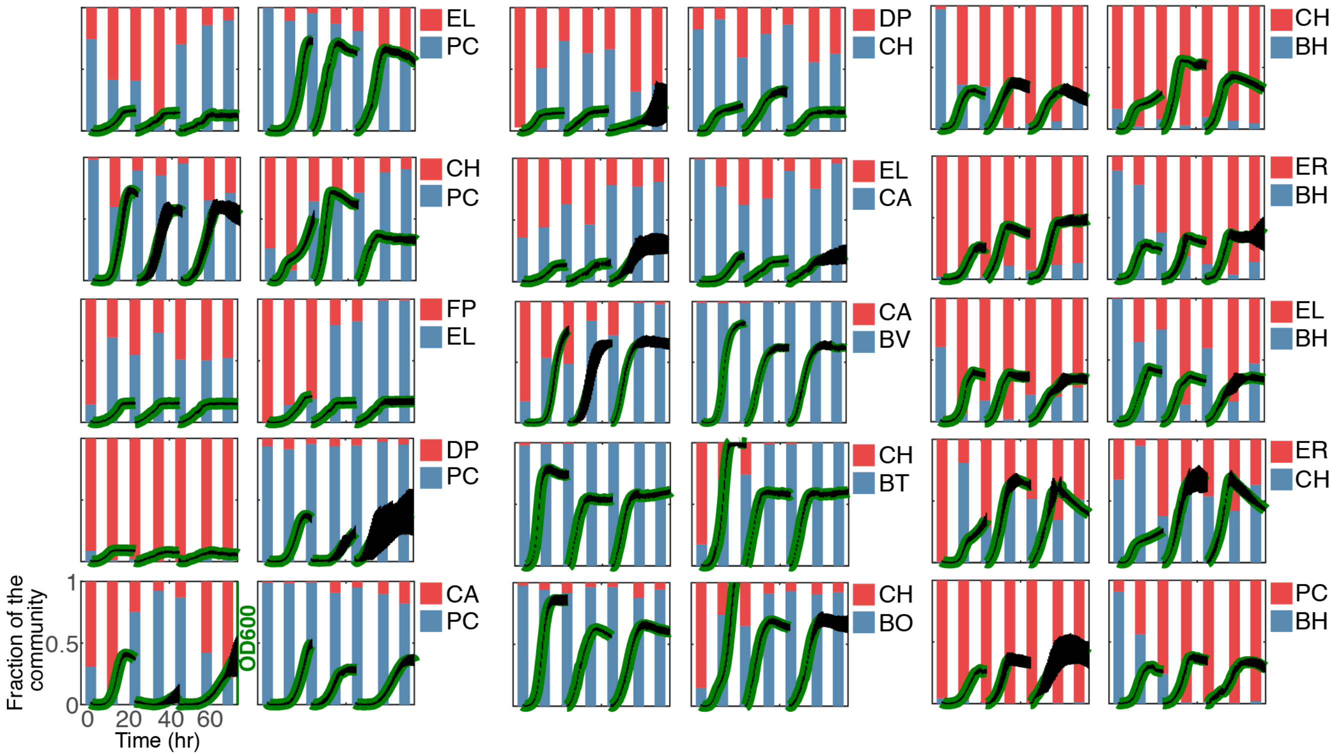

Appendix Figure S4

Time-series measurements of relative and absolute abundance based on OD600 measurements of 15 pairwise communities inoculated using two different initial species proportions: 95% species A, 5% species B and the reciprocal based on OD600 values. Each subplot shows time (x-axis) vs. fraction of the community (left y-axis) and OD600 (right y-axis). Error bars denote 1 s.d. from the mean of at least three biological replicates.

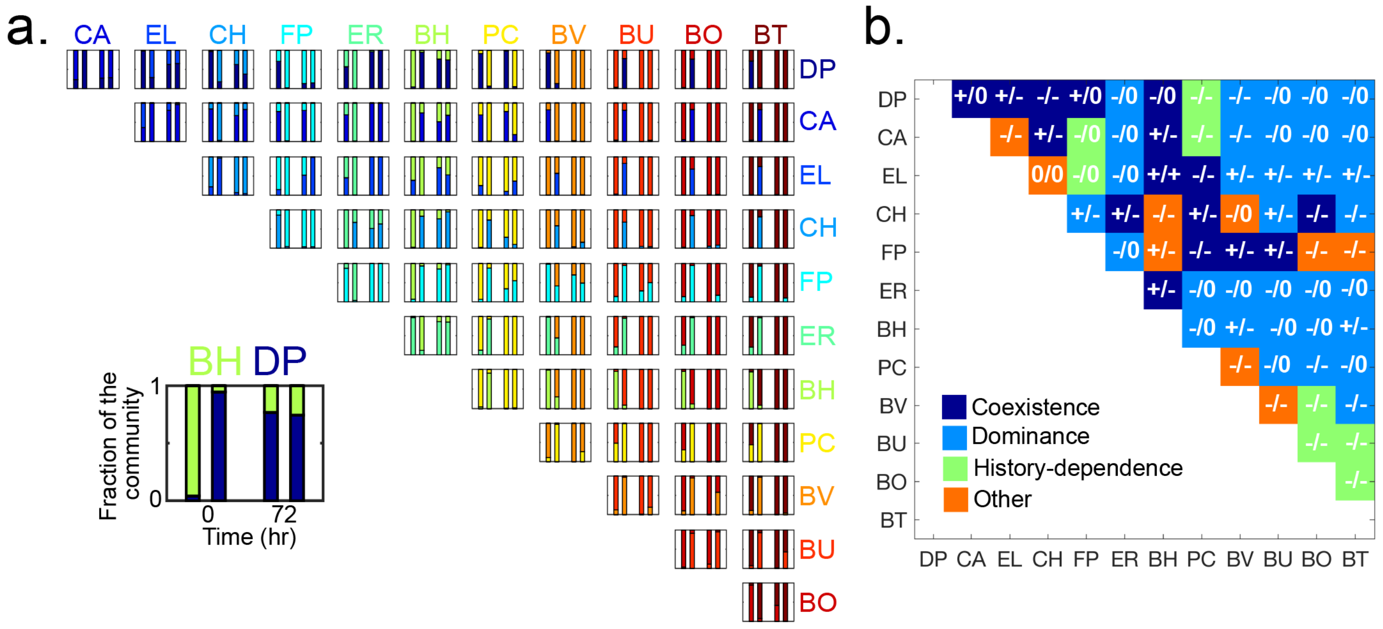

Appendix Figure S5

Classification of community behaviors based on consortia inoculated using two distinct initial species proportions. (a) Relative abundance of pairwise assemblages inoculated from two distinct initial species proportions based on OD600 measurements: 95% species A, 5% species B and a second wherein these percentages were reversed. Community composition was measured immediately following inoculation (t = 0)

and following 72 hr using the serial dilution experimental design. (b) Heat-map of pairwise community behaviors classified into *coexistence*, *dominance*, *history-dependence* or *other* categories. *History-dependence* required at least 40% variation in relative abundance at 72 hr for each organism based on two conditions inoculated using distinct initial species proportions. *Dominance* occurred when one organism was at least 95% of the community at 72 hr and the community did not exhibit a history-dependent response. *Coexistence* required that both species were at least 5% of the community at 72 hr and the consortium did not exhibit a history-dependent response. Communities in the *other* category did not quantitatively satisfy the criteria for *history-dependence*, *dominance* or *coexistence*. The text in each box represents the signs of the inferred inter-species interactions for the model trained on T3.

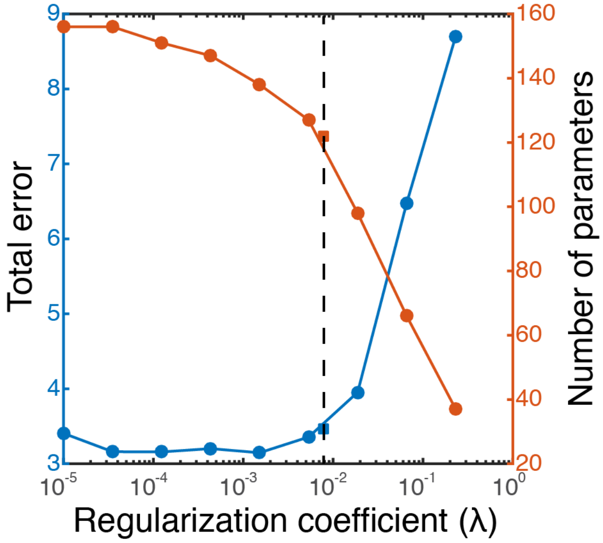

Appendix Figure S6

Relationship between the regularization coefficient (λ), sum of errors (total error) for all experiments in the T3 training set (left y-axis) and number of parameters with an absolute value greater than 0.01 (right y-axis). The total error was computed as the sum of mean squared errors for each organism in each community (see Materials & Methods). A high-resolution scan of λ yielded an optimal value corresponding to λ = 0.0077 (dashed line).

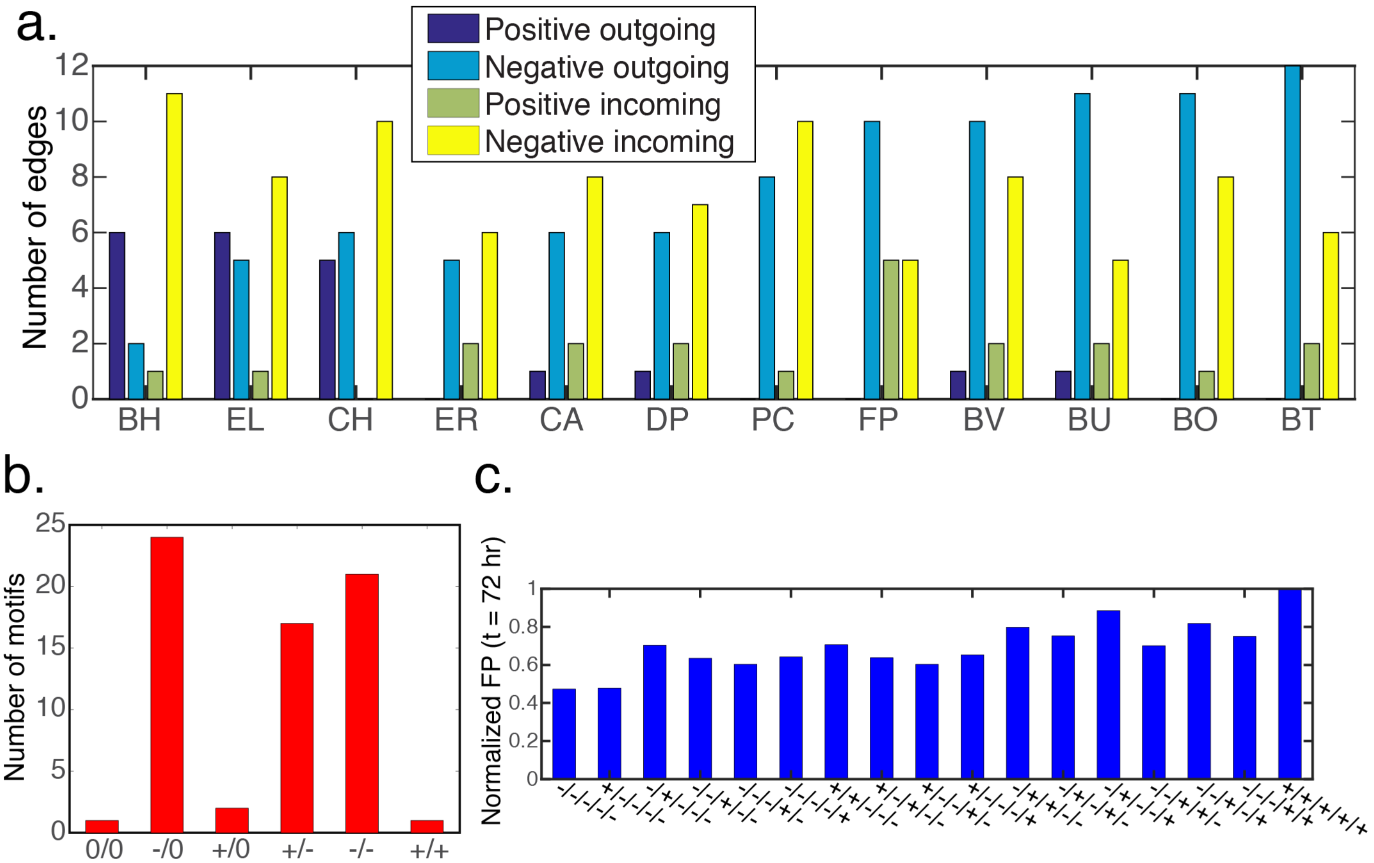

Appendix Figure S7
Analysis of the inferred gLV inter-species interaction coefficient network based on training set T3. (a) Bar plot of the number of positive and negative incoming and outgoing edges for each organism for the inferred network based on the T3 training set. (b) Bar plot of the total number of qualitatively distinct network topologies in the inferred gLV model trained on T3. (c) Normalized absolute abundance of FP to the maximum value across parameter sets at 72 hr in a 6-member gLV model composed of FP and positive modulators of FP: BH, BU, BV, CH and DP. A '+' or '-' indicates that the incoming inter-species interaction coefficient for FP $\alpha_{FP,X}$ was set to the inferred value based on training set T3 or zero, respectively. Here X represents BH, BU, BV, CH, and DP, respectively.

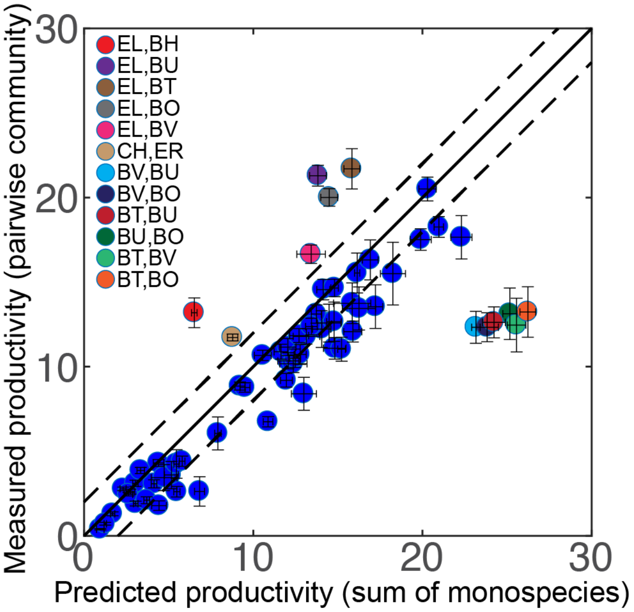

Appendix Figure S8.
Scatter plot of predicted vs. measured productivity for all pairwise communities. Productivity is defined as the integral of the growth response based on OD600 as a function of time. Predicted productivity is a null model of the sum of integrals of monospecies growth responses based on OD600 as a function of time. Measured productivity is computed as the integral of the pairwise community growth response over a 23.5 hr period. The solid and dashed lines represent the x = y line and two-fold significance threshold, respectively. Error bars represent 1 s.d. from the mean of at least three biological replicates.

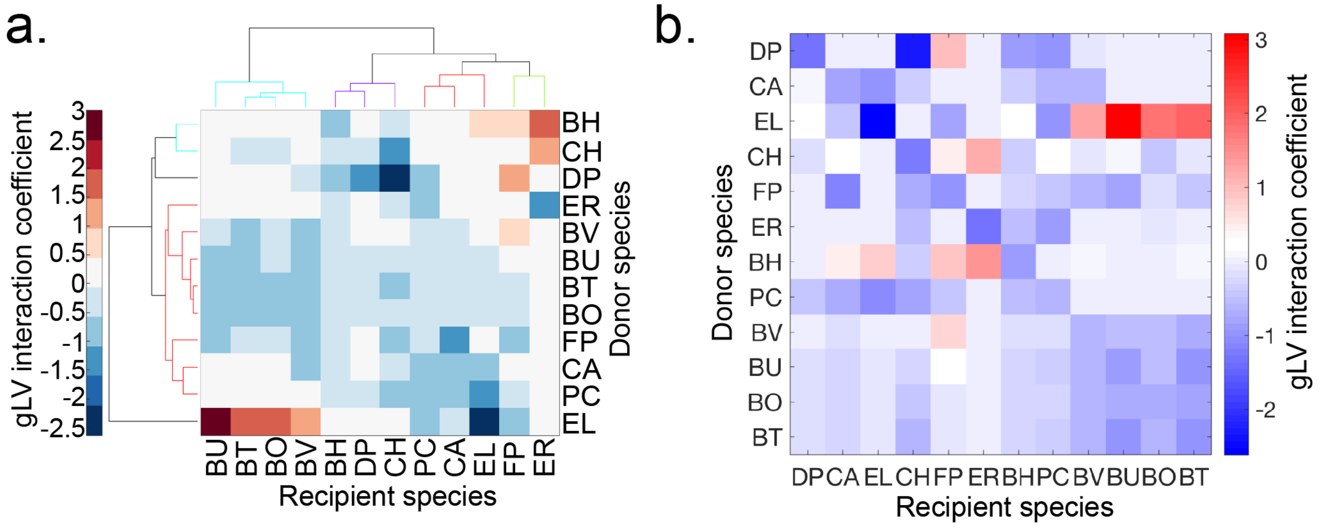

Appendix Figure S9
Analysis of the patterns in the inferred inter-species interaction coefficients for the gLV model trained on T3 based on phylogenetic relatedness. (a) Hierarchical clustering of inferred inter-species interaction coefficients for the model trained on T3. Recipient and donor species are on the x and y-axis, respectively. The color of the heat-map represents the value of the gLV interaction coefficient. (b) Heat-map of inter-species interaction coefficients sorted by phylogenetic relatedness for the gLV model trained on T3. Recipient and donor species are on the x and y-axes, respectively.

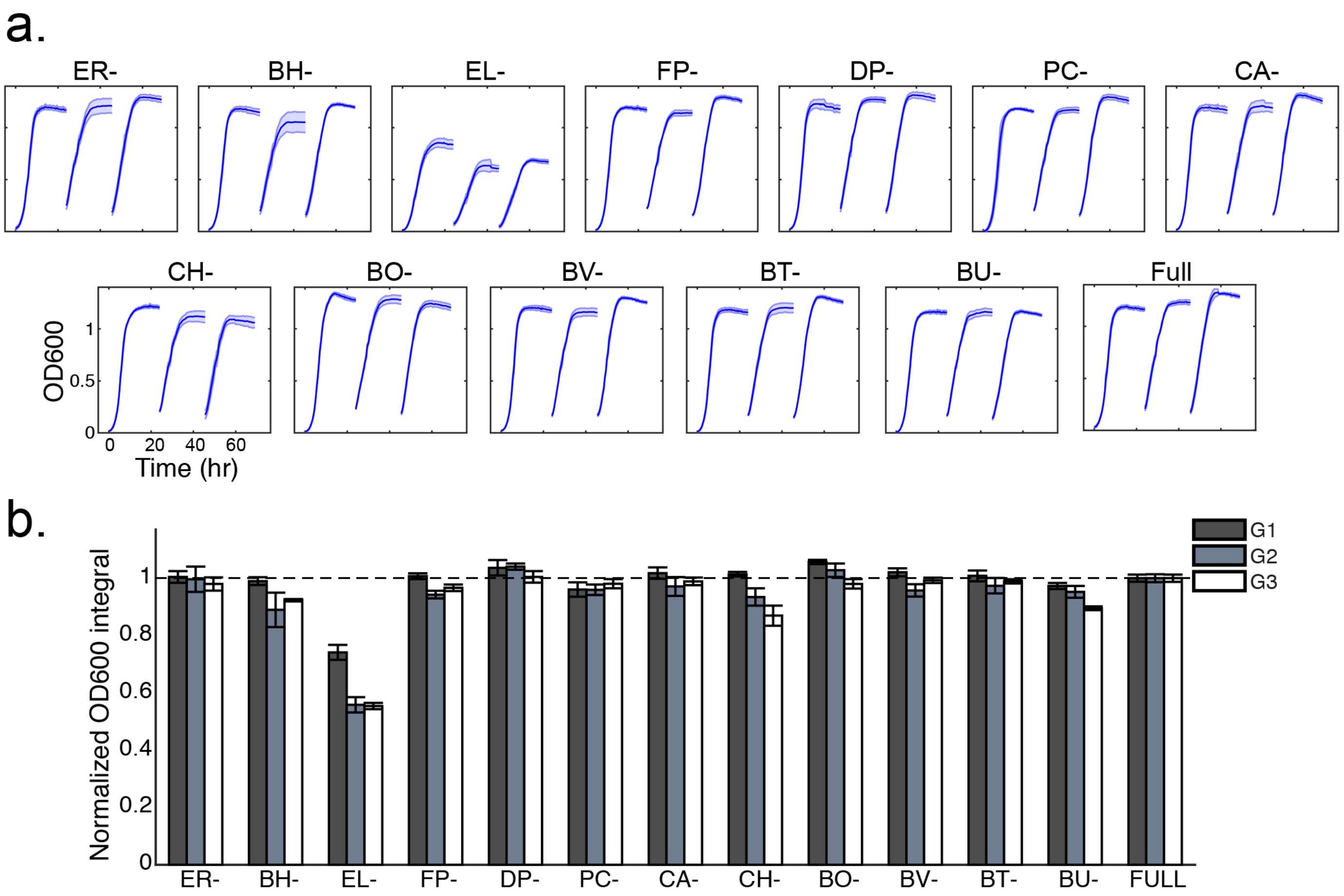

Appendix Figure S10

Growth responses of multi-species communities as a function of time. (a) Total biomass was quantified using absorbance at 600 nm (OD600) for the full (12-member) and single-species communities (11-member). Each subplot represents a single species dropout or the full community. Text above each subplot indicates the organism absent from the community. Shaded regions denote 1 s.d. from the mean of six biological replicates. (b) Normalized integral of each time-resolved growth response for each multi-species community. Growth stages G1-G3 represent the growth response of the community following inoculation and after the first and second serial transfers (1:20 dilution), respectively. Error bars represent 1 s.d. from the mean of six biological replicates.

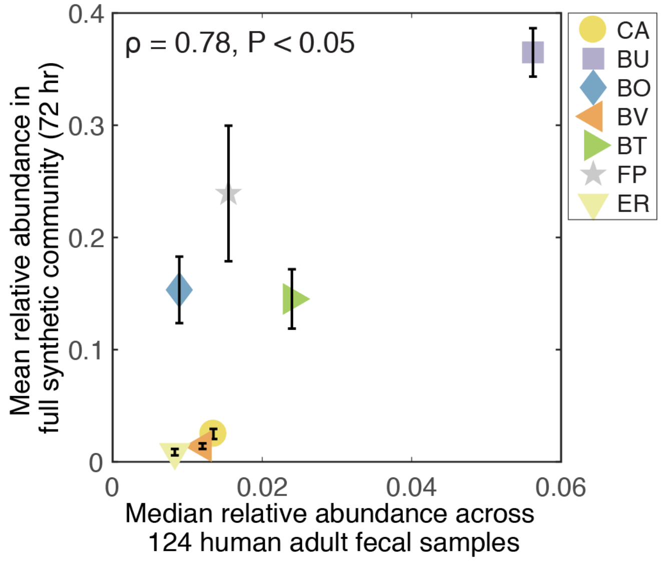

Appendix Figure S11
Scatter plot of the median relative abundance across 124 adults based on metagenomics sequencing(Qin *et al*, 2010) vs. the mean relative abundance of each species in the full synthetic community at 72 hr. Error bars represent 1 s.d. from the mean of six biological replicates. ρ and P denote the Pearson correlation coefficient and p-value, respectively.

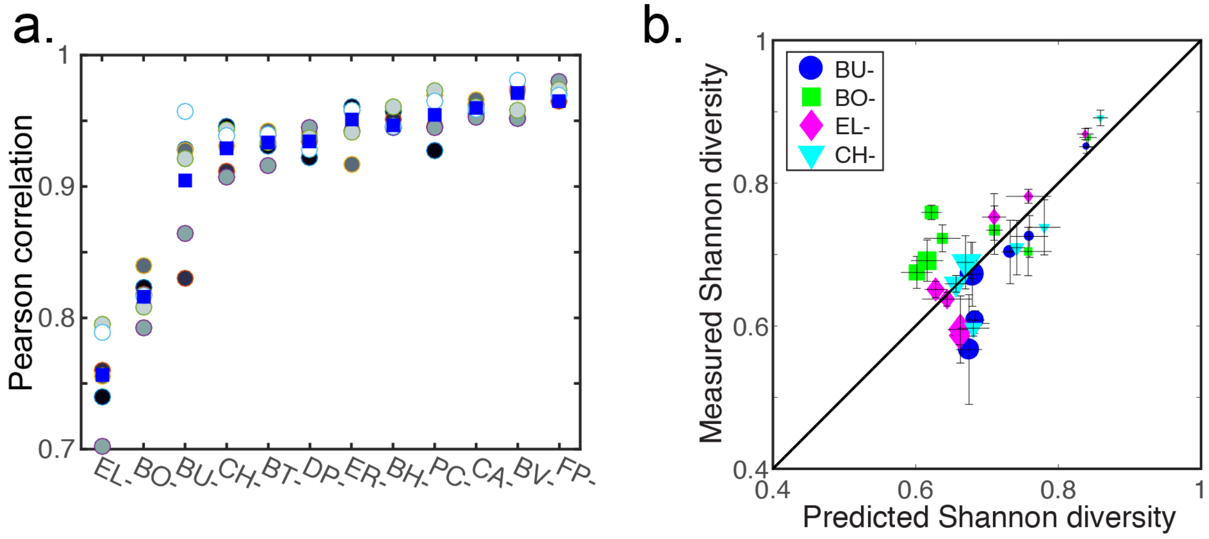

Appendix Figure S12
Evaluating the contribution of each organism to community assembly based on time-series measurements of community structure of multi-species consortia. (a) Average Pearson correlation coefficient between a renormalized full community containing only the set of shared species and the single-species dropout consortium for all time points. $\hat{v}$ denotes the renormalized relative abundance of the shared species between the full (12-member) and single-species dropout communities. Here $\hat{v}_i = \frac{v_i}{\sum_{i \in C} v_i}$ where C denotes the set of 11 shared organisms between the full and single-species dropout community. Data points represent biological replicates (circles) and mean of six biological replicates (blue squares), respectively. (b) Scatter plot of community diversity scored by the Shannon equitability index. Predicted Shannon diversity represents the renormalized full community consisting of the shared set of species with the single-species dropout consortium. Measured Shannon diversity denotes the Shannon equitability index for the single-species dropout communities EL-, BU-, BO- or CH-. Community diversity was computed using the Shannon equitability index: $E_H = \frac{\sum_{i=1}^{n} p_i \ln p_i}{\ln S}$ where S and p_i denote the total number of species and relative abundance of each organism,

respectively. The size of the data point is proportional to the time point of sample measurement. Error bars represent 1 s.d. from the mean of six biological replicates.

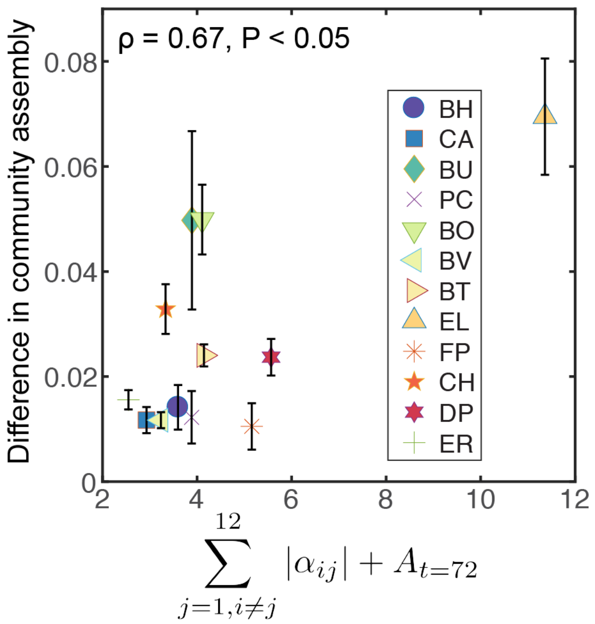

Appendix Figure S13

Scatter plot of the species impact score vs. the difference in community assembly for single-species dropout consortia. The species impact score is defined as $\sum_{j=1, i\neq j}^{12} |\alpha_{ij}| + A_{t=72}$ where α_{ij} represents the inferred inter-species interaction coefficients for the gLV model trained on T3 and $A_{t=72}$ denotes the relative abundance of each species in the full community at 72 hr. Difference in community assembly was computed as $\sum_{i=1,}^{11} \frac{1}{7} \sum_{j=1}^{7} (\hat{v}_{ji,FULL} - v_{ji,X})^2$ where i and j represent species and time points, respectively. X represents a single-organism dropout community lacking the single organism X. $\hat{v}$ represents the renormalized relative abundance of the shared set of species in the full (12-member) and single-species dropout communities. Here $\hat{v}_i = \frac{v_i}{\sum_{i\in C} v_i}$ where C denotes the set of 11 shared organisms in the full and single-species dropout community. ρ and P denote the Pearson correlation coefficient and p-value, respectively. Error bars represent 1 s.d. from the mean of six biological replicates.

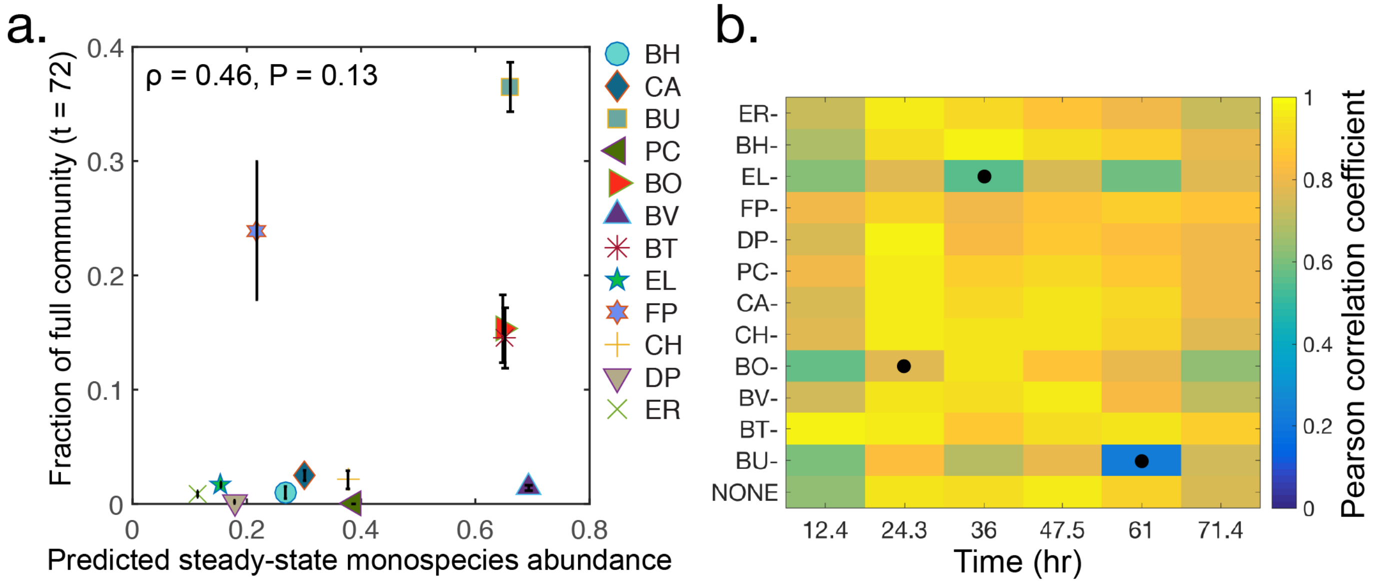

Appendix Figure S14

(a) Scatter plot of the predicted steady-state monospecies abundance using the gLV model trained on T3 vs. the relative abundance of each species in the full community at 72 hr. The predicted steady-state monospecies concentration was computed as $x_e = -\mu_i \alpha_{ii}$ where μ_i and α_{ii} represent the growth rate and intra-species interaction coefficient, respectively. Error bars represent 1 s.d. from the mean of six biological replicates. ρ and P denote the Pearson correlation coefficient and p-value, respectively. (b) Heat-map of Pearson correlation coefficients across time (x-axis) and multi-species communities (y-axis). Circles (black) denote conditions that were not statistically significant. Pearson correlations were statistically significant ($P < 0.05$) except for the EL- at 36 hr ($P = 0.062$), BU- at 61 hr ($P = 0.47$) and BO- at 24.3 hr ($P = 0.051$).

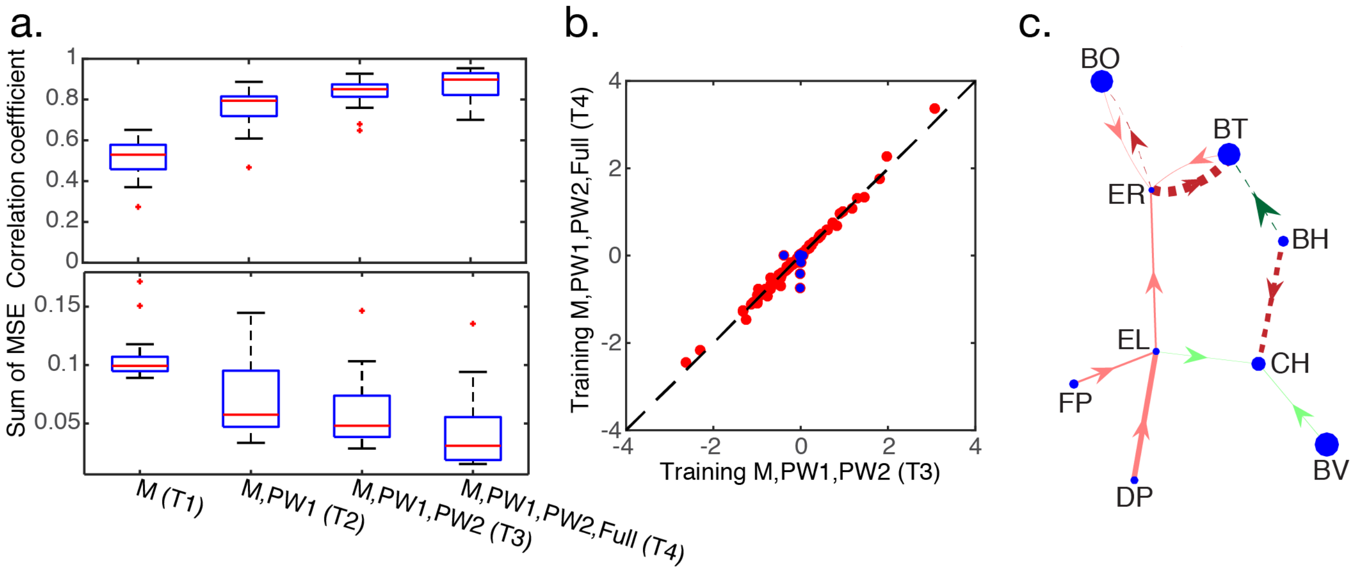

Appendix Figure S15

The model trained on T4 exhibited the highest predictive accuracy of temporal changes in multi-species community structures. (a) Metrics to evaluate the predictive accuracy of the models include the Pearson correlation coefficient (top) and sum of mean squared errors across all species in each community (bottom, see Materials & Methods). The x-axis represents model training sets. The validation data set for T1-T3 included 12 single-species dropouts (11-member communities) and the full community (12-member) and T4 was validated on the set of 11-member consortia. On each box, the red line represents the median, the edges of the box are the 25th and 75th percentiles, the whiskers extend to the most extreme data points and the outliers are plotted as red crosses. (b) Scatter plot of inferred parameters for gLV models trained on T3 vs. T4.

Blue data points represent parameters that were significantly different in the model trained on T4 compared to T3. (c) Network diagram of inter-species interaction coefficients that were significantly different in models trained on T4 compared to T3. Edges represent interaction coefficients that were present in T4 and absent in T3 (solid lines) or absent in T4 and present in T3 (dashed lines). Red and pink lines denote negative interactions and dark and light green represent positive interactions. Node size and line width are proportional to the steady-state abundance of monospecies and magnitude of inter-species interaction coefficients, respectively.

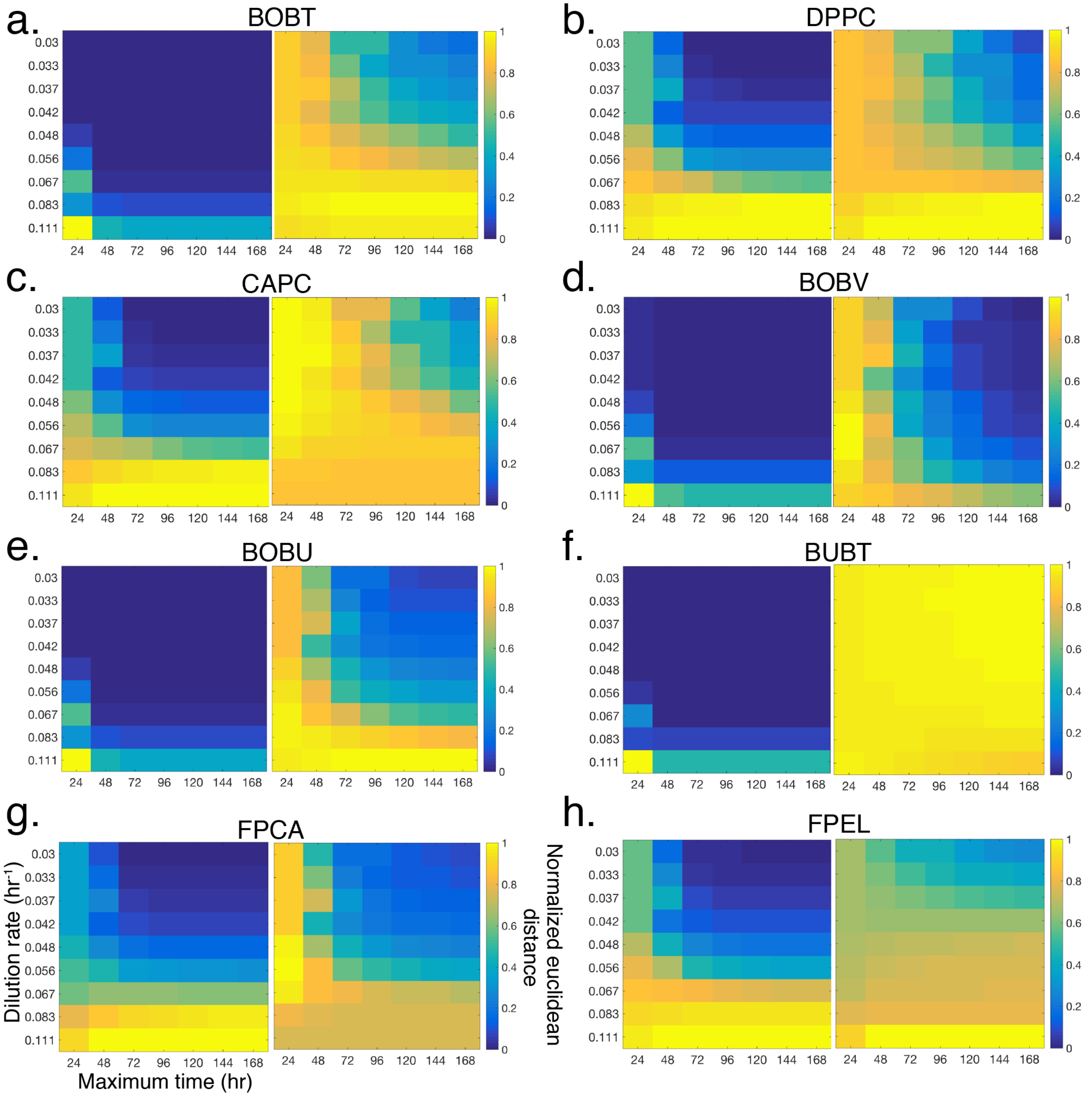

Appendix Figure S16

Model analysis of pairwise community history-dependent responses across a broad range of simulated serial dilution rates. History-dependent responses were evaluated based on the Euclidean distance of species concentrations from an equilibrium point for the maximum number of serial dilutions within the time period. Each panel shows the Euclidean distance from the equilibrium point normalized to the maximum value in the absence (α_{ij}, α_{ji} = 0, left) and presence (right) of inferred inter-species interactions for the gLV model trained on

T3. The x and y-axes represent time periods and dilution rates, respectively. Pairwise communities include (a) BO, BT; (b) DP, PC; (c) CA, PC; (d) BO, BV; (e) BO, BU; (f) BU, BT; (g) FP, CA and (h) FP, EL. Monostable communities include BO, BT; DP, PC; CA, PC; BO, BV; BO, BU, FP, CA; FP, EL and BU, BT exhibited bistability.

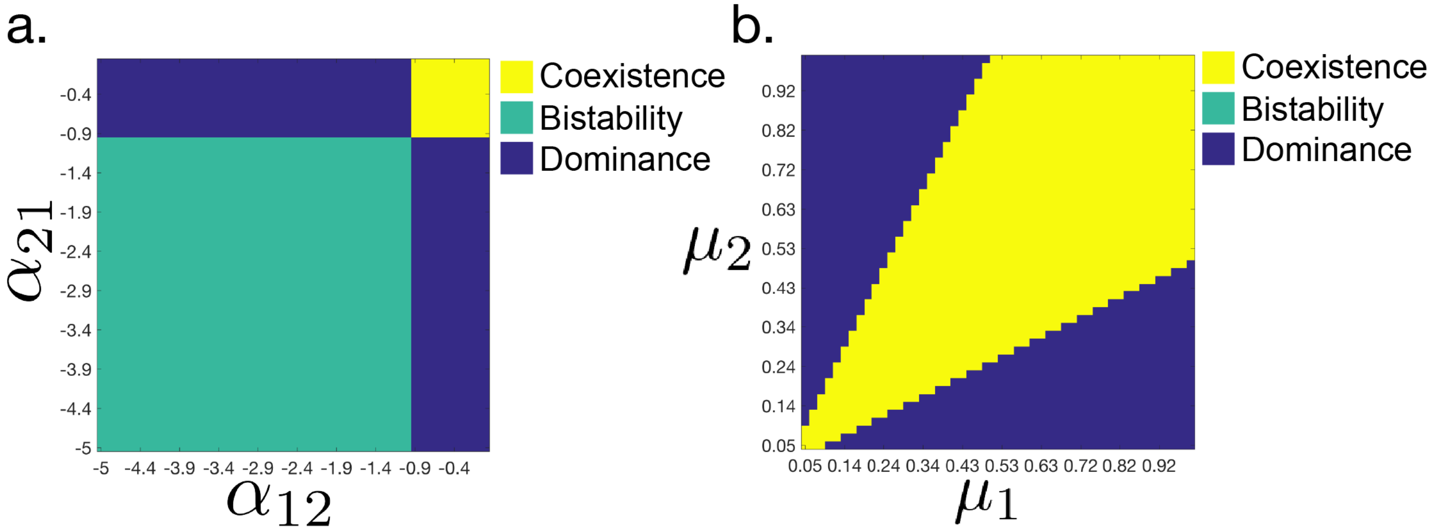

Appendix Figure S17

Bifurcation diagrams for a pairwise community coupled by bidirectional negative inter-species interactions. (a) Bifurcation diagram showing parameter regimes for coexistence, bistability and single-species dominance across a range of inter-species interaction coefficient values (α_{ij}). Parameters included μ_1 = 0.1, μ_2 = 0.1, α_{11} = -1 and α_{22} = -1. (b) Bifurcation diagram of steady-state behaviors as a function of growth rate parameters (μ_i). Parameters included α_{11} = -1, α_{22} = -1, α_{21} = -0.5 and α_{12} = -0.5.

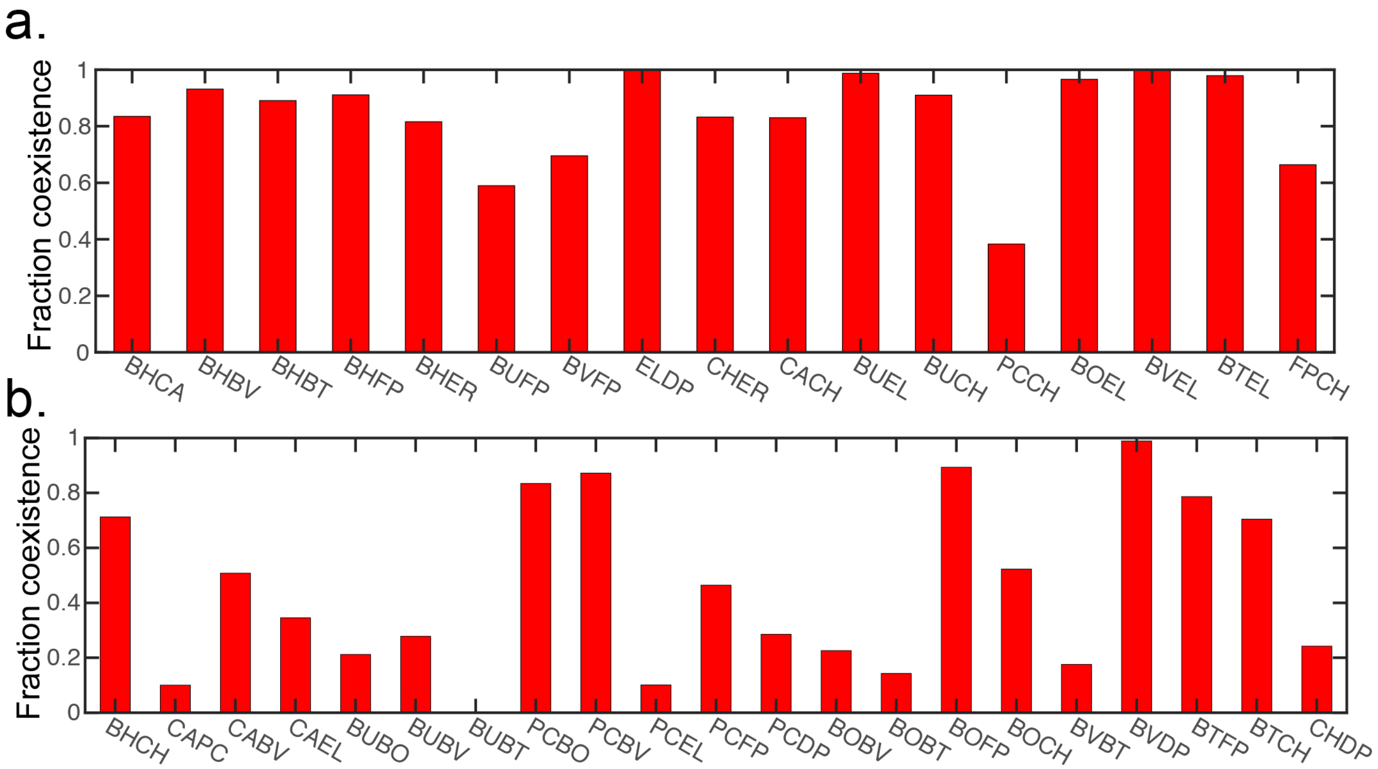

Appendix Figure S18.

Fraction of parameter space that exhibits species coexistence across a broad range of growth rates in the model. Networks included all inferred (a) coupled positive/negative or (b) bidirectional negative topologies for the gLV model trained on T3. Coexistence was determined by computing the analytical steady-state solution of

the pairwise gLV model for each parameter set. Growth rate parameters ranged from 0.05 to 1 hr^{-1} and 2500 combinations of growth rate parameters within this range were evaluated.

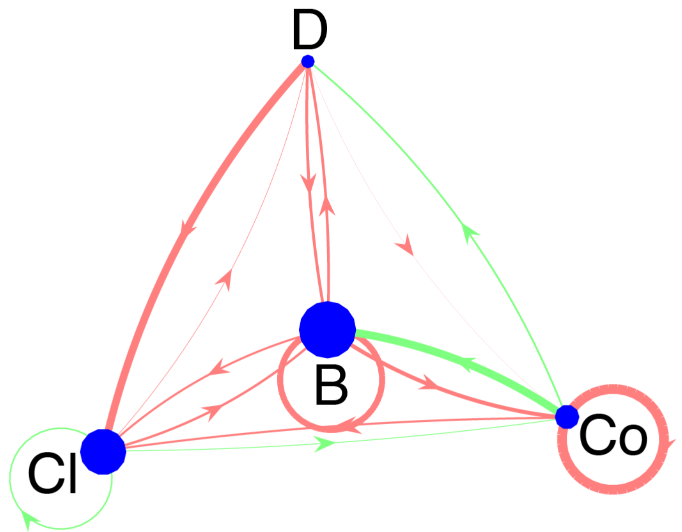

Appendix Figure S19

Order-level interaction network for the gLV model trained on T3. Cl, D, B and Co denote *Clostridiales* (FP, BH, ER and CH), *Desulfovibrionales* (DP), *Bacteroidales* (PC, BV, BU, BT and BO) and *Coriobacteriaceae* (CA and EL), respectively. Green and red edges represent averaged positive and negative inter-species interaction coefficients across all species associated with the given order and line width represents the magnitude of the averaged interaction. Node size denotes the number of species in each order in the synthetic community.

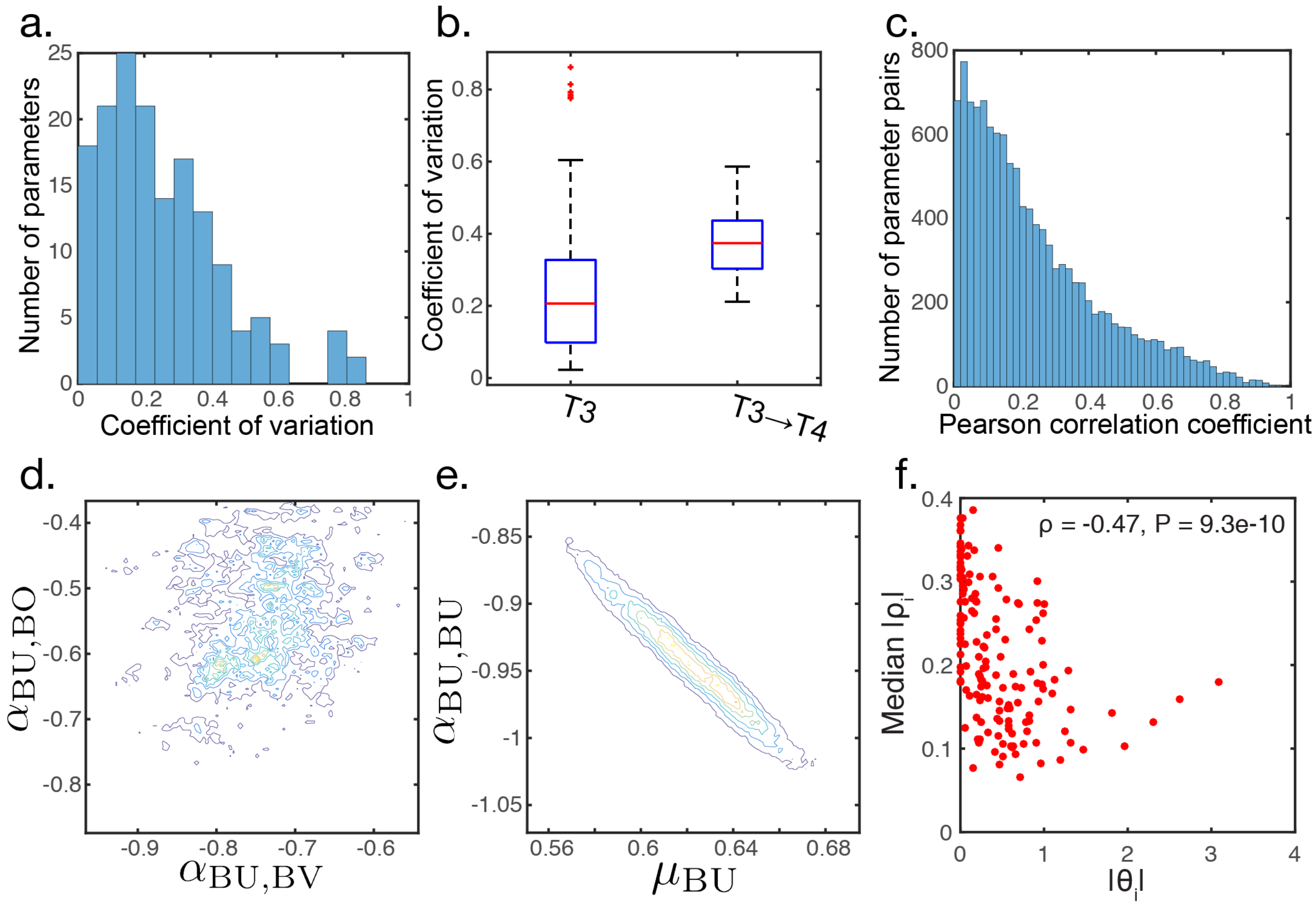

Appendix Figure S20

Metropolis-Hastings Markov Chain Monte Carlo (MCMC) analysis of parameter uncertainty for the gLV model trained on T3. Results were based on a single Markov chain that was run for 670000 iterations and used a burn-in period of 100000 iterations. The chain was initialized from the inferred parameter set based on T3. (a) Histogram of the coefficient of variation (CV) of the parameters for 570000 iterations. (b) Box plot of the CV of inter-species interaction coefficients that were not significantly different between model training on T3 vs. T4 (145 parameters) and coefficients that were significantly different based on training on T3 vs. T4 (T3→T4). On each box, the red line represents the median, the edges of the box are the 25th and 75th percentiles, the whiskers extend to the most extreme data points that the algorithm considers not to be outliers, and the outliers are plotted as red crosses. (c) Histogram of the absolute value of the Pearson correlation (ρ) for all parameter pairs for 570000 iterations. (d) Representative contour plot of the covariation between $\alpha_{BU,BV}$ and $\alpha_{BU,BO}$ (ρ = 0.23). (e) Representative contour plot of a highly correlated parameter pair μ_{BU} and $\alpha_{BU,BU}$ (ρ = 0.97). (f) Scatter plot of the absolute value of each inferred parameter for the gLV model trained on T3 vs. the median absolute value of the Pearson correlation based on Metropolis-Hastings MCMC. ρ and P denote the Pearson correlation coefficient and P-value, respectively.

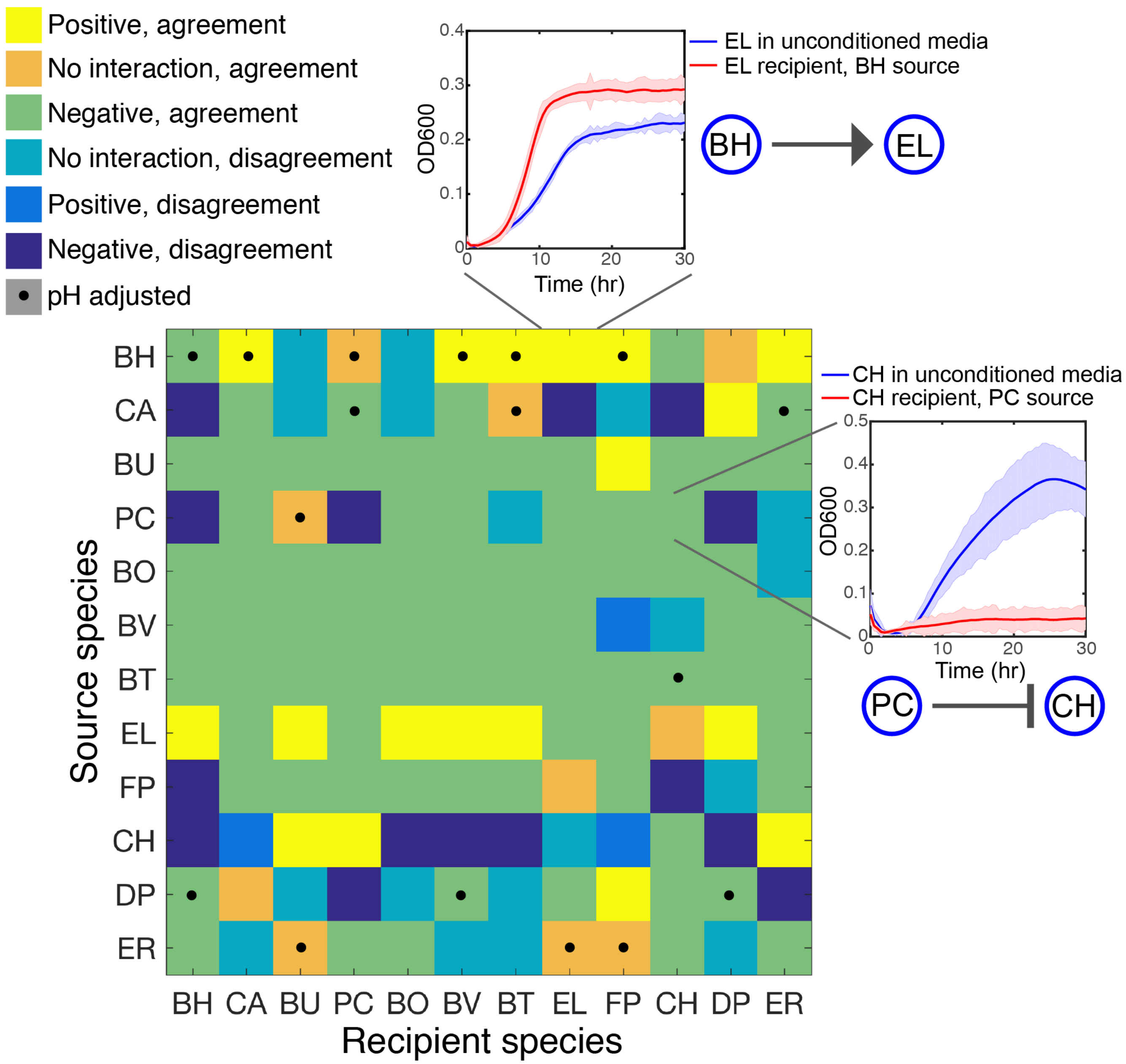

Appendix Figure S21

Qualitative comparison between the impact of conditioned media on monospecies growth and the sign of the inferred gLV interaction coefficients for the gLV model trained on T3. Recipient species (x-axis) were exposed

to 75% conditioned media from a source organism (y-axis). The cumulative sum of the recipient growth response over a 30 hr period was computed in the presence of conditioned media, unconditioned media and pH adjusted conditioned media wherein the pH of the conditioned media was modified to approximately equal the pH of the unconditioned media. The ratio of the cumulative sum of the growth response in conditioned media (R_{CM}) or pH adjusted conditioned media (R_{CMp}) to the unconditioned media was compared to the sign of the gLV interaction coefficient. The results were in agreement if the R_{CMp}, R_{CM} > 1 and α_{ij} > 1e-6 (positive interaction) or R_{CMp}, R_{CM} < 1 and α_{ij} < -1e-6 (negative interaction) or 0.9 < R_{CMp}, R_{CM} < 1.1 and $|\alpha_{ij}| \leq$ 1e-6 (no interaction). Insets: representative growth responses of CH in the presence and absence of PC conditioned media (negative interaction) and EL in the presence and absence of BH conditioned media (positive interaction). Shaded regions denote 1 s.d. from the mean (solid lines) of four biological replicates.

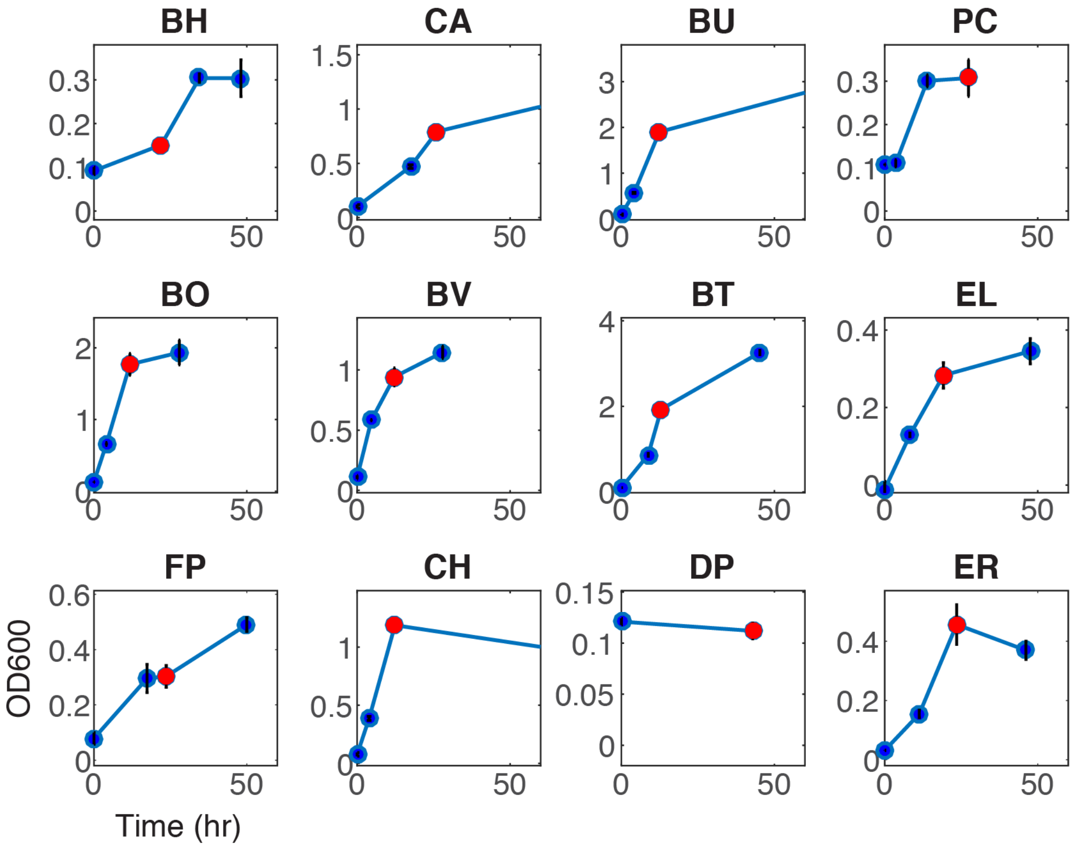

Appendix Figure S22
OD600 measurements as a function of time for monospecies cultures used for exo-metabolomic profiling. The red data point represents the OD600 value at the time of the exo-metabolomics measurement. Error bars represent 1 s.d. from the mean of three biological replicates.

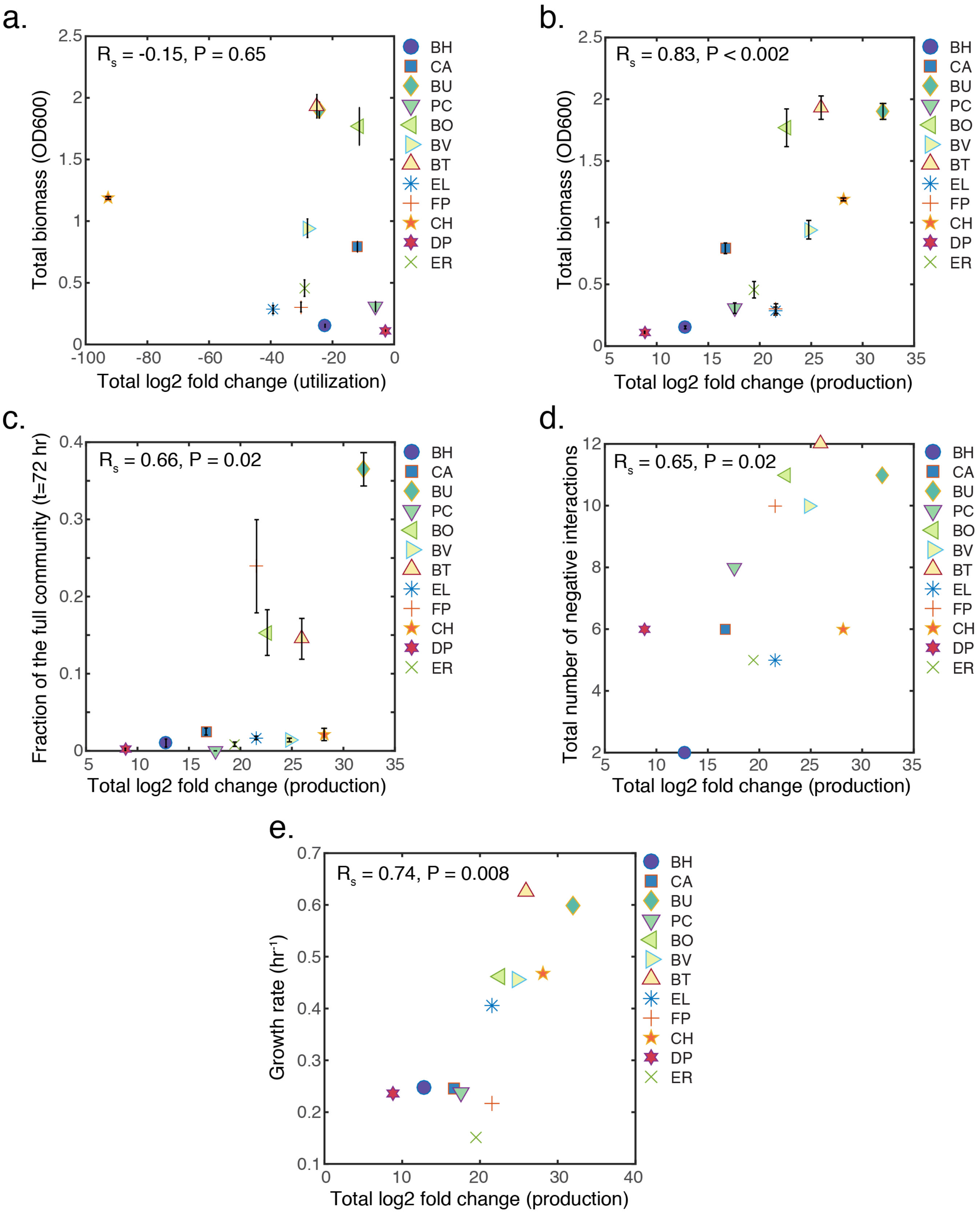

Appendix Figure S23

Relationships between log2 fold change in metabolite abundances, monospecies biomass yields, community structure and inferred negative interactions for the gLV model trained on T3. (a) Scatter plot of the total log2 fold change in metabolite utilization vs. monospecies biomass at the corresponding time point. Error bars represent 1 s.d. from the mean of three biological replicates. (b) Scatter plot of the total log2 fold change in

metabolite secretion vs. monospecies biomass at the corresponding time point. Error bars represent 1 s.d. from the mean of three biological replicates. (c) Scatter plot of the total log2 fold change in metabolite secretion vs. species relative abundance in the full community at 72 hr. Error bars represent 1 s.d. from the mean of six biological replicates. (d) Scatter plot of the total log2 fold change in metabolite secretion vs. total number of negative interactions in the inferred gLV model trained on T3. (e) Scatter plot of the total log2 fold change in metabolite secretion vs. the growth rate of monospecies in the gLV model trained on T3. R_s and P denote the Spearman rank correlation coefficient and p-value, respectively.

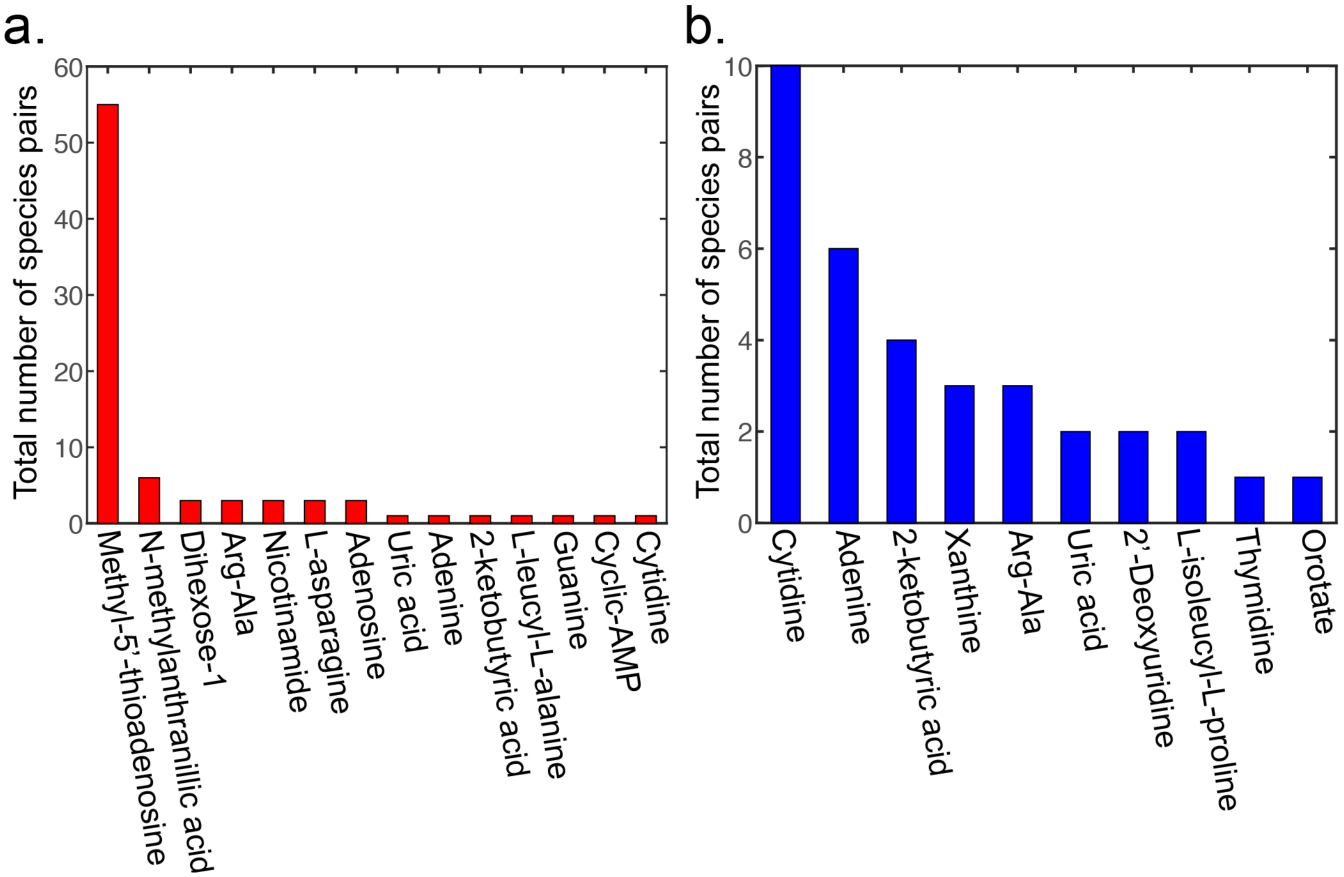

Appendix Figure S24

Bar plots of metabolites predicted to mediate negative or positive interactions in the synthetic community based on exo-metabolomic profiling. (a) Number of species pairs predicted to co-consume metabolites leading to a potential negative interaction. Consumed metabolites decreased by at least two-fold relative to the beginning of the experiment. (b) Number of species pairs predicted to consume a metabolite that was secreted by a different organism. Consumed and secreted metabolites decreased or increased by at least two-fold relative to the beginning of the experiment, respectively.

	BH	CA	BU	PC	BO	BV	BT	EL	FP	CH	DP	ER
μ	0.297	0.345	0.769	0.126	0.733	0.583	0.878	0.171	0.162	0.710	0.208	0.090
BH	-0.790	0.000	0.000	0.000	0.000	0.000	0.000	0.000	0.000	0.000	0.000	0.000
CA	0.000	-0.784	0.000	0.000	0.000	0.000	0.000	0.000	0.000	0.000	0.000	0.000
BU	0.000	0.000	-1.121	0.000	0.000	0.000	0.000	0.000	0.000	0.000	0.000	0.000
PC	0.000	0.000	0.000	-0.515	0.000	0.000	0.000	0.000	0.000	0.000	0.000	0.000
BO	0.000	0.000	0.000	0.000	-1.082	0.000	0.000	0.000	0.000	0.000	0.000	0.000
BV	0.000	0.000	0.000	0.000	0.000	-0.754	0.000	0.000	0.000	0.000	0.000	0.000
BT	0.000	0.000	0.000	0.000	0.000	0.000	-1.164	0.000	0.000	0.000	0.000	0.000
EL	0.000	0.000	0.000	0.000	0.000	0.000	0.000	-0.646	0.000	0.000	0.000	0.000
FP	0.000	0.000	0.000	0.000	0.000	0.000	0.000	0.000	-0.522	0.000	0.000	0.000
CH	0.000	0.000	0.000	0.000	0.000	0.000	0.000	0.000	0.000	-1.492	0.000	0.000
DP	0.000	0.000	0.000	0.000	0.000	0.000	0.000	0.000	0.000	0.000	-0.655	0.000
ER	0.000	0.000	0.000	0.000	0.000	0.000	0.000	0.000	0.000	0.000	0.000	-0.558

Appendix Figure S25. Table of inferred growth rates and interaction coefficients for the gLV model trained on T1 (M). Donor and recipient species are listed in rows and columns, respectively. μ represents monospecies growth rate.

	BH	CA	BU	PC	BO	BV	BT	EL	FP	CH	DP	ER
μ	0.199	0.264	0.637	0.214	0.513	0.528	0.679	0.477	0.214	0.629	0.192	0.107
BH	-0.802	0.309	0.000	0.186	0.000	0.264	0.000	0.790	0.882	0.000	0.000	0.000
CA	-0.162	-0.854	0.000	0.403	0.000	-0.064	0.000	-0.002	0.000	-0.383	0.090	0.000
BU	-0.149	-0.290	-0.967	-0.253	-0.568	-0.684	-1.067	-0.028	0.183	-0.269	-0.110	0.000
PC	-0.002	-0.552	0.000	-0.872	-0.002	0.000	0.000	0.000	-0.504	-0.498	0.000	0.000
BO	-0.141	-0.312	-0.777	-0.182	-0.810	-0.671	-0.894	-0.051	-0.114	0.235	-0.124	0.001
BV	-0.054	-0.138	-0.577	-0.119	-0.547	-0.786	-0.761	0.000	0.656	0.000	-0.022	0.001
BT	-0.183	-0.298	-0.980	-0.232	-0.639	-0.717	-1.030	-0.055	-0.071	-0.716	-0.132	0.000
EL	1.132	-0.339	2.949	-0.169	1.924	1.558	1.792	-3.423	-0.761	0.000	0.201	0.000
FP	-0.071	-0.902	-0.867	0.000	-0.172	-0.750	-0.552	0.000	-0.946	-0.855	0.000	0.000
CH	-0.067	0.276	0.051	0.360	-0.560	-0.037	0.000	0.000	0.385	-1.740	-0.124	0.862
DP	-0.528	0.000	0.000	0.000	0.000	-0.002	0.000	0.000	0.945	0.000	-0.892	0.000
ER	0.000	0.000	0.000	0.000	0.000	0.000	0.000	0.000	0.000	-0.515	0.000	-0.717

Appendix Figure S26. Table of inferred growth rates and interaction coefficients for the gLV model trained on T2 (M and PW1). Donor and recipient species are listed in rows and columns, respectively. μ represents monospecies growth rate.

	BH	CA	BU	PC	BO	BV	BT	EL	FP	CH	DP	ER
μ	0.247	0.246	0.599	0.238	0.462	0.456	0.626	0.405	0.216	0.468	0.236	0.151
BH	-0.924	0.430	0.000	0.000	0.000	0.134	0.058	0.829	0.909	-0.385	0.000	1.471
CA	-0.312	-0.818	0.000	-0.583	0.000	-0.654	0.000	-0.993	0.000	-0.319	0.053	0.000
BU	-0.228	-0.261	-0.906	-0.324	-0.506	-0.577	-0.972	-0.078	0.199	-0.216	-0.163	-0.028
PC	-0.529	-0.685	0.000	-0.615	0.000	0.000	0.000	-1.091	-0.423	-0.784	-0.441	0.000
BO	-0.220	-0.283	-0.749	-0.272	-0.713	-0.577	-0.824	-0.093	-0.114	-0.468	-0.187	0.000
BV	-0.131	-0.170	-0.558	-0.204	-0.509	-0.658	-0.682	-0.023	0.725	0.000	-0.017	-0.018
BT	-0.264	-0.274	-0.938	-0.304	-0.577	-0.613	-0.960	-0.099	-0.074	-0.591	-0.192	-0.031
EL	0.182	-0.477	3.082	-0.985	1.810	1.285	1.967	-2.620	-0.800	0.000	0.199	0.000
FP	-0.237	-1.121	-0.831	-0.451	-0.176	-0.635	-0.450	0.000	-0.995	-0.694	0.000	-0.032
CH	-0.328	0.303	0.056	0.241	-0.467	-0.046	-0.073	0.000	0.431	-1.242	-0.147	1.191
DP	-0.918	0.000	0.000	-1.001	0.000	-0.108	0.000	0.000	0.976	-2.301	-1.323	0.000
ER	-0.549	0.000	0.000	-0.916	-0.039	0.000	0.000	0.000	0.000	-0.508	0.000	-1.322

Appendix Figure S27. Table of inferred growth rates and interaction coefficients for the gLV model trained on T3 (M, PW1 and PW2). Donor and recipient species are listed in rows and columns, respectively. μ represents monospecies growth rate.

	BH	CA	BU	PC	BO	BV	BT	EL	FP	CH	DP	ER
μ	0.245	0.246	0.584	0.237	0.478	0.457	0.598	0.402	0.219	0.502	0.232	0.156
BH	-0.912	0.453	0.000	0.000	0.000	0.137	0.000	0.692	0.961	0.000	0.000	1.343
CA	-0.306	-0.829	0.000	-0.560	0.000	-0.657	0.000	-1.098	0.000	-0.241	0.042	0.000
BU	-0.228	-0.261	-0.880	-0.324	-0.632	-0.584	-0.754	-0.124	0.231	-0.151	-0.176	-0.061
PC	-0.529	-0.671	0.000	-0.622	0.000	0.000	0.000	-1.077	-0.402	-0.771	-0.433	0.000
BO	-0.215	-0.278	-0.921	-0.265	-0.734	-0.556	-0.819	-0.114	-0.099	-0.465	-0.203	-0.020
BV	-0.129	-0.168	-0.550	-0.202	-0.515	-0.660	-0.755	-0.049	0.759	0.039	-0.026	-0.030
BT	-0.272	-0.274	-0.816	-0.303	-0.624	-0.642	-0.907	-0.169	-0.069	-0.632	-0.200	0.000
EL	0.176	-0.448	3.378	-0.904	1.757	1.303	2.272	-2.442	-0.768	0.024	0.176	-0.139
FP	-0.231	-1.121	-0.782	-0.405	-0.209	-0.638	-0.703	-0.153	-1.038	-0.507	0.000	-0.168
CH	-0.348	0.312	0.066	0.265	-0.507	-0.047	-0.086	0.000	0.448	-1.454	-0.151	1.083
DP	-0.900	0.000	0.000	-0.977	0.000	-0.106	0.000	-0.408	1.015	-2.157	-1.254	0.000
ER	-0.546	0.000	0.000	-0.817	0.000	0.000	-0.738	0.000	0.000	-0.439	0.000	-1.271

Appendix Figure S28. Table of inferred growth rates and interaction coefficients for the gLV model trained on T4 (M, PW1, PW2 and full community). Donor and recipient species are listed in rows and columns, respectively. μ represents monospecies growth rate.

REFERENCES

Qin J, Li R, Raes J, Arumugam M, Burgdorf KS, Manichanh C, Nielsen T, Pons N, Levenez F, Yamada T, Mende DR, Li J, Xu J, Li S, Li D, Cao J, Wang B, Liang H, Zheng H, Xie Y, et al (2010) A human gut microbial gene catalogue established by metagenomic sequencing. *Nature* **464:** 59–65
